# Specialty Grand Challenge In Pediatric Infectious Diseases

**DOI:** 10.3389/fped.2017.00185

**Published:** 2017-08-28

**Authors:** Philippe Lepage, Sophie Blumental

**Affiliations:** ^1^Hôpital Universitaire des Enfants Reine Fabiola, Brussels, Belgium; ^2^Université Libre de Bruxelles (ULB), Brussels, Belgium

**Keywords:** vaccination, multiresistant pathogens, pediatric infectious diseases, emerging infectious diseases, tropical diseases

It’s tough to make predictions, especially about the future.

(Yogi Berra, famous baseball player)

## Introduction

In recent years, decrease in global mortality and improvement in quality of life have been among the most significant progresses in child and adult health (1). In the field of pediatric infectious diseases, remarkable advances have been achieved in terms of reduction of incidence of childhood infectious diseases and associated morbidity and mortality. This is especially true for vaccine-preventable diseases where immunization against measles (2), *Haemophilus influenzae* type B (3), pneumococci (4, 5), meningococci (6), rotavirus (7), varicella (8), hepatitis B virus (HBV) infection (9, 10), etc., has dramatically decreased the burden of targeted infections in high- and low-income countries. The global challenge of controlling HBV dissemination well illustrates how preventive measures could hinder the serious course of an infectious disease. Indeed, in the case of HBV infection, perinatal exposure represents an important mode of transmission leading to chronic disease in ~90% of infected newborns. Following its implementation in many countries, the universal vaccination of babies born from HBV positive mothers has considerably modified the epidemiology of HBV infection worldwide (9). When given the best immunoprophylaxis currently available (hepatitis B vaccine and hepatitis B immune globulins), perinatal HBV infection only occurs among ~1% of infants of infected mothers (10). The success obtained from HBV vaccine as well as from many others vaccination campaigns led to consider prevention by life-saving immunizations as one of the seven greatest achievements in pediatric research observed during the last four decades, as emphasized by a recent review (1). In addition to vaccination and prenatal screening, other preventive measures such as improvement of environmental conditions and personal hygiene together with broadening supply in drinkable water also significantly contributed to reduce prevalence and burden of highly transmissible infections (i.e., cholera, shigellosis, or hepatitis A) in different parts of the world. The old principle issued from Chinese traditional medicine “An ounce of prevention is worth a pound of cure” achieves its real significance when thinking about pediatric infectious disease. Again, the recent success story of preventing mother-to-child transmission of human immunodeficiency virus (HIV) well illustrates this keystone concept. In high prevalence regions, universal HIV testing of pregnant women combined with the use of antiretroviral therapy but also with avoidance of breastfeeding and scheduled cesarean section in mothers at high risk of transmission permitted all together to decrease the risk of perinatal transmission to <2% in many countries (11) with subsequent impact on childhood mortality.

On the other hand, the management of common pediatric infectious diseases has also been completely modified during the last decades aiming to increase therapeutic efficacy but also to lower related complications and societal cost. A great proportion of pneumonia (12) and urinary tract infections (13) are now treated with a very short inpatient stay or even fully on ambulatory basis, instead of the long and painful intravenous therapeutic courses previously recommended. Even deep-seated infections such as septic arthritis and osteomyelitis, formerly associated with extended periods of hospitalization, are managed now, in most instances, through oral ambulatory treatment after a short 3–5 days hospital stay (14, 15). Along with new drugs available, assessment of pediatric pharmacokinetic (PK) and pharmacodynamic (PD) drugs properties and significant insights in diagnostic procedures, the trend is now to shorten hospital stay to improve patient’s comfort, decrease risk of secondary nosocomial infections and avoid overwhelming hospital costs.

Frontiers in Pediatrics/Pediatric Infectious Diseases section will prioritize the abovementioned concepts but also aims to look forward and anticipate some future challenges that Pediatric Infectious Diseases specialists will have to face in the next 10–20 years. Our main goal will be to guide and encourage researchers to submit papers dealing with present and future critical areas of research, as outlined below.

## Vaccination

Despite the success of many immunization programs detailed above, caveats and disparity in our vaccinations policies remain. First, as emphasized in a recent World Health Organization Report (16), nearly one in five children worldwide did not receive routine immunization against vaccine-preventable diseases leading to unacceptable inequality. Besides political and economic issues, providing immunization all over the world will require further development on less expensive and thermostable vaccine formulations, as recently achieved for a low-cost, heat-stable oral bovine pentavalent rotavirus vaccine produced in India and tested in Sub-Saharan Africa (17, 18). Among infants in Niger, its efficacy after a three doses schedule against severe rotavirus gastroenteritis was comparable to that measured with current licensed rotavirus vaccines in developing countries (17). Second, more time and funding should be dedicated to develop vaccines against pathogens considered as ubiquitous human killers but for which existing vaccines remain disappointing in terms of effectiveness [influenza, pertussis, tuberculosis, or malaria (19)]. New candidate vaccines against emerging diseases such as Zika virus, Ebola virus, Chikungunya virus, and Middle East respiratory syndrome coronavirus (MERS-CoV) infections are also needed. Whereas these latter are close to be evaluated in humans (20), convincing results will be required among adults first before being tested in pediatric subjects. Third, on a population scale, the key role of herd immunity in contributing to vaccine effectiveness combined with dynamic circulation of bacteria renders mandatory continuous evaluations of vaccines impact on diseases epidemiology. This is especially true when looking at the “replacement phenomenon” (switch from bacterial serotypes to others) observed inside the nasopharyngeal niche after universal mass vaccination by pneumococcal conjugate vaccines (21). Fourth, maternal immunization represents an expanding field of investigation with numerous new scientific hypotheses to be investigated (22, 23) (see below, next section). Eventually, besides its expected conventional effects on diseases incidence and prevalence, some authors recently highlighted that vaccination also produces non-specific beneficial immunological effects (24, 25). Indeed, strong scientific evidences suggest that certain live attenuated vaccines (BCG or measles vaccines) can reduce all-cause mortality, probably through conferring some cross-protection against additional untargeted pathogens (24). This surprising and fascinating hypothesis definitely deserves further studies to be elucidated. For all these reasons, experts from Johns Hopkins and Yale Universities classify pediatric immunization as one of the next seven great priorities in pediatric research (26).

## The Return of Old Diseases

We are facing, in some parts of the world, the return of old diseases such as pertussis (27), tuberculosis (28), and measles (29) for which finding innovative strategies in prevention and therapy is essential. In recent years, efforts to limit the familial spread of pertussis to young infants have been developed in addition to maternal immunization and providing to adolescents and adults an additional booster dose coupled with diphtheria–tetanus. However, efficient cocooning strategies against pertussis are difficult to implement and neonatal vaccination has limitations. For example, defining the optimal timing of maternal immunization against pertussis remains controversial (30, 31). As for measles, its prevalence in Europe has decreased by >90%, but the disease is far from being eradicated and now reemerged in many countries with numerous outbreaks associated sometimes with life-threatening presentations (29). Therefore, improved clinical awareness combined with epidemiological and virological surveillance using modern laboratory tools and efficient reporting systems are required for global eradication (2, 29).

## The Emergence of Multiresistant Pathogens

The emergence of multiresistant bacteria (Gram-positive cocci, Gram-negative rods, MDR tuberculosis, XDR tuberculosis, etc.) is a major global threat. During the past 4 years, many countries reported for the first time arrival of carbapenemases producing rods (32). Among others, New Delhi metallo-β-lactamase 1 has been isolated from *Klebsiella pneumoniae* and *Escherichia coli* worldwide, offering few treatment opportunities when these pathogens are involved in invasive diseases (33). Whereas this issue already constitutes a major concern in adult setting, data about epidemiology and optimal treatment options are even more limited in pediatrics. To avoid permanent extrapolation from adult data, we need well-conducted pediatric studies assessing the impact of various treatment strategies. As performed in adult setting (34), such kind of studies will help us to address crucial unanswered questions like what is the most suitable antibiotic options for a defined pathogen, which dosage use according to age groups, what is optimal duration of therapy, etc. Furthermore, the problem of resistance of fungal and viral infections should not be neglected and will increase in parallel with the broader use of immunosuppressive therapies and advances in bone marrow and solid organ transplants. New antiviral drugs against CMV or adenovirus infections have been recently developed and can be obtained in some instances on compassionate use, offering a last chance for some patients suffering from a disseminated uncontrolled viral disease (35). Again, data in children remain limited to some case series but these new drugs seem promising and their use is worth to be reported. In parallel, significant insights in specific immunotherapy to treat disseminated CMV, adenovirus, or EBV infections in transplanted patients offer an encouraging reliable approach to improve the prognosis of these serious illnesses (36).

## Clinical Trials in Pediatric Infectious Diseases

As mentioned in the above section, proper pediatric clinical trials with large sample sizes should be encouraged rather than always basing management of pediatric patients on extrapolation from adult studies. This is all the more important in the field of pharmacology of antimicrobials. Still too many drugs, including last available antimicrobials, are brought on the market without any pediatric-specific labeling or PK:PD data and lack reliable pediatric recommendations (26). It is common knowledge that drug metabolism differs among age groups, not only because of body surface but also because of variations in renal and hepatic drug excretion. Therefore, finding the optimal therapeutic dosage which offers a maximum safety and efficacy is challenging in children and particularly in neonates, even when following the results from blood drug monitoring (37). Huge differences exist when looking for example at metabolism and excretion of antifungals in newborns and infants compared to adults. Broader clinical trials are therefore warranted to determine the adequate dosages in each age group, especially in the youngest for which in addition major antifungals like voriconazole and posaconazole remain not indicated due to lack of safety data (38) restricting therapeutic options in case of invasive central nervous system fungal infection. Likewise, only few small pediatric studies assess the best dosage of new antibiotics like moxifloxacin or linezolid, and only few papers provide suitable data to guide our choice among the vast antibiotics armamentarium (39). More studies in children are needed that will compare the efficiency of different antibiotic schedules on morbidity, mortality, or in preventing recurrences of infections caused by common susceptible bacteria (e.g., MSSA) (40).

## Emerging Infectious Diseases

The field of emerging infectious diseases (MERS-CoV, Enterovirus D68, Zika, Ebola, Chikungunya, etc.) is also of utmost importance. Recent outbreaks have created significant important threats in certain regions of the world and required important mobilization of resources and health-care workers, in providing care to infected peoples as well as in developing diagnostic tools, new drugs, and vaccines. In the setting of a new outbreak, making the right clinical diagnosis is crucial, not only for the patient himself but also because it will allow for management of the epidemics through rapid confinement of infected people. Therefore, publication of reliable epidemiological data based on wide surveillance programs and description of all encountered clinical patterns are top priorities. Enterovirus D68 infection has recently emerged as a major viral agent in some regions of the world and, although this virus was considered as mostly benign, quality clinical and laboratory studies delineate now its place as a serious respiratory and central nervous system pathogen (41, 42). Besides common cold or spontaneously resolving meningitis, some infected children indeed presented with acute respiratory failure requiring ICU admission, and others developed acute flaccid paralysis mimicking the pattern observed in polio virus infection (42). What this viral infection will elicit in the near future definitely deserves further careful surveillance, especially since neither antiviral drugs nor vaccine against this virus are currently available.

## Pediatric Infectious Diseases and Immunology

Recent advances in pediatric research have dramatically improved the survival of immunocompromised children and adolescents (43) However, whether immunosuppression is acquired or congenital, infectious diseases remain a major cause of morbidity and mortality in this particular population and improving the management of fungal and viral infections in children represents a top priority. During the last 30 years, exciting discoveries on genetic mechanisms underlying rare primary immunodeficiencies have been carried out (44) leading to better understanding of the extraordinary vulnerability presented by some patients to specific pathogens (non-tuberculous mycobacteria, *Salmonellae* sp., etc.) because of Mendelian and non-Mendelian transmitted genetic syndromes (44). No doubt that this field of investigation—requiring the collaboration of immunologists, geneticians, and pediatric infectious diseases specialists—will continue to grow at a rapid pace during the following years, especially regarding the exponential development of genome sequencing methods over the last decade. As of 1st August 2017, more than 150 primary immunodeficiencies have been identified, corresponding to 357 genes mutations (45). Since the deficient pathways underlying these syndromes are every day increasingly understood and since significant insights are made in gene therapy and bone marrow transplant, new hopes arise for the management of these rare life-threatening diseases.

## Recognizing the Role of Inflammation

New primary immunodeficiency syndromes were recently described in patients, which presented not only with recurrent life-threatening infections but also with auto-inflammatory manifestations or neurodevelopmental delay. In these specific syndromes linked for example to a defect or a gain of function in interferon pathways (46, 47), the auto-inflammatory pattern can be the inaugural or even unique feature of the disease and seriously impairs patient’s growth and quality of life.

Beside the field of congenital immunodeficiency, scientists increasingly recognize the inflammation cascade as playing a crucial role in the symptomatology harbored by infected patients. This modern concept sustains whether disproportionate immune host responses can generate significant damages in addition to those expected from the pathogen itself. Even looking a paradox at first glance, controlling the inflammation in these cases should be integrant part of the therapy together with antimicrobials, to avoid treatment failure and poor outcome.

The immune reconstitution syndrome observed in HIV-infected patients well illustrates this concept. Indeed, a worsening of clinical course was observed when antiretroviral therapy was started too early during a severe mycobacterial or fungal infectious episode (48). Likewise, sound studies now point out the key role of inflammation in morbidity and mortality related to a broad range of infections like tuberculous meningitis (49), cystic fibrosis (50), and meningococcal invasive disease (51).

## Tropical Diseases

The burden of tropical diseases (malaria, schistosomiasis, Chagas disease, neglected tropical diseases, etc.) is substantial and will remain so for many years, despite progresses in vaccination, environmental preventive measures and available treatments. Whereas their impact obviously peaks in low-income countries, tropical diseases are also more and more encountered in non-endemic countries where, as a consequence of global migration, a growing proportion of patients are originated from endemic regions (immigrants or refugees) or are relatives visiting friends in such parts of the world, in addition to touristic travelers (52). Therefore, good knowledge of the epidemiology and therapies of these diseases is mandatory for all clinicians from both tropical and non-tropical areas. Recently, the use of artemisinin-based combination therapy (ACT) has considerably improved the management of malaria both in adults and children (52). However, artemisinin resistance occurs in Southeast Asia (53), and late treatment failures with ACT have been reported in non-immune European subjects due to subtherapeutic drug concentrations (54).

## The Balance Between Technological Progresses and Clinical Epidemiology

Technological progresses in the field of pediatric infectious diseases have been tremendous: we are now able, for example, to look for numerous pathogens in a single small respiratory sample thanks to DNA amplification by molecular biology (55). Recent progresses in biomolecular technology have allowed, among others, rapid detection of mutations conferring antibiotics resistance, efficient genotyping methods or even sequencing of the whole genome of virulent organisms like MRSA (56), ESBL Gram-negative bacteria (57) and *Mycobacterium tuberculosis* (58); all of these opening the door to new targeted therapeutics and preventive strategies as well as better surveillance in case of epidemics (dissemination of clusters, epidemic curves, etc.).

Although important in modern medicine, technological advances are not the only answer to all the challenges we are facing. Wide knowledge of semiology, clinicians constant awareness, good prescription of diagnostic tools, and appropriate infection control measures are more than ever crucial in our era to place the patient at the center of our preoccupations and to guarantee a rational use of all available resources. Far from being old fashioned, quality clinical and epidemiological studies are needed, using basic tools to monitor trends in diseases incidence and anticipate future epidemics. Excellent studies carried out in the 1960s and 1970s on the clinical epidemiology of viral pathogens causing common respiratory infections (respiratory syncytial virus, influenza, adenovirus, etc.) in Chapel Hill, North Carolina, are still precious and inspiring in our daily practice (59, 60).

## Conclusion

In conclusion, keeping in mind this non-exhaustive lecture, no doubt there will be many exciting initiatives and areas of investigation worth of attention in the near future. We also definitively need new approaches to implement research and disseminate knowledge so that science and technological advances will be translated into policies easily available for people who need them the most (26). The renewed Editorial Board of Frontiers in Pediatrics/Pediatric Infectious Diseases is now ready to launch and discuss new opportunities of scientific research with you and looks forward to receive your suggestions in this field.

## Author Contributions

All authors listed have made a substantial, direct, and intellectual contribution to the work and approved it for publication.

## Conflict of Interest Statement

The authors declare that the research was conducted in the absence of any commercial or financial relationships that could be construed as a potential conflict of interest.
